# Impact of adjuvant hormonotherapy on radiation-induced breast fibrosis according to the individual radiosensitivity: results of a multicenter prospective French trial

**DOI:** 10.18632/oncotarget.24606

**Published:** 2018-03-02

**Authors:** Céline Bourgier, Florence Castan, Olivier Riou, Tan-Dat Nguyen, Karine Peignaux, Claire Lemanski, Jean-Léon Lagrange, Youlia Kirova, Eric Lartigau, Yazid Belkacemi, Sofia Rivera, Georges Noël, Sébastien Clippe, Françoise Mornex, Christophe Hennequin, Sophie Gourgou, Muriel Brengues, Pascal Fenoglietto, Esat Mahmut Ozsahin, David Azria

**Affiliations:** ^1^ Institute de Recherche en Cancérologie de Montpellier, Inserm U1194, Université de Montpellier, Institut Régional du Cancer de Montpellier, Montpellier, France; ^2^ Institute Jean Godinot, Reims, France; ^3^ Centre GF Leclerc, Dijon, France; ^4^ AP-HP Henri Mondor, Créteil, France; ^5^ Institute Curie, Paris, France; ^6^ Centre Oscar Lambret, Lille, France; ^7^ Gustave Roussy, Villejuif, France; ^8^ Centre Paul Strauss, Strasbourg, France; ^9^ Centre Marie Curie, Valence, France; ^10^ Centre Hospitalier Lyon Sud, Pierre Bénite, France; ^11^ AP-HP Saint-Louis, Paris, France; ^12^ Centre Hospitalier Universitaire Vaudois, Lausanne, Switzerland

**Keywords:** radiotherapy, breast cancer, hormonotherapy, individual radiosensitivity, late effects

## Abstract

**Background:**

To evaluate risk of severe breast fibrosis occurrence in patients treated by breast-conserving surgery, adjuvant radiotherapy and hormonotherapy (HT) according to individual radiosensitivity (RILA assay).

**Results:**

HT^–^ and RILA^high^ were the two independent factors associated with improved breast-fibrosis free survival (BFFS). BFFS rate at 36 months was lower in patients with RILA^low^ and HT^+^ than in patients with RILA^high^ and HT^–^ (75.8% and 100%, respectively; *p* = 0.004, hazard ratio 5.84 [95% confidence interval (CI) 1.8–19.1]). Conversely, BFFS at 36 months was comparable in patients with RILA^high^ and HT^+^ and in patients with RILA^low^ and HT^–^ (89.8% and 93.5%, respectively; *p* = 0.39, hazard ratio 1.7 [95% CI 0.51–5.65]), showing that these two parameters influenced independently the occurrence of severe breast fibrosis. BFFS rate was not affected by the HT type (tamoxifen or aromatase inhibitor) and timing (concomitant or sequential with radiotherapy).

**Conclusions:**

HT and RILA score independently influenced BFFS rate at 36 months. Patients with RILA^high^ and HT^–^ presented an excellent BFFS at 36 months (100%).

**Materials and methods:**

Breast Fibrosis-Free Survival (BFFS) rate was assessed relative to RILA categories and to adjuvant HT use (HT^+^ and HT^–^, respectively) in a prospective multicentre study (NCT00893035) which enrolled 502 breast cancer patients (456 evaluable patients). Breast fibrosis was recorded according to CTCAE v3.0 grading scale; RILA score was defined according to two categories (<12%: RILA^low^; ≥12%: RILA^high^).

## INTRODUCTION

The efficacy of adjuvant breast cancer radiotherapy after breast-conserving surgery is now well established. The Early Breast Cancer Trialists’ Collaborative Group (EBCTCG) meta-analysis showed that the addition of radiotherapy significantly reduces breast cancer recurrence and death [1]. In addition to radiotherapy, endocrine treatment (tamoxifen, TAM, and/or aromatase inhibitors, AI) also significantly decreases all recurrences and the 10-year mortality rate for breast cancer [2].

However, late side effects have been reported in long-term survivor patients with better breast cancer outcome. These toxicities, such as radio-induced cardiotoxicity or poor cosmetic outcome, could impair the quality of life and affect the clinical benefit over time [3, 4]. The risk factors of normal tissue radiosensitivity are related to the personal medical history and/or directly to the treatment [5]. It has been hypothesized that severe fibrosis occurs mostly in patients with micro-vascularization diseases (e.g., diabetes mellitus, hypertension, etc.) or with diseases related to excess collagen deposition (e.g., scleroderma). Other risk factors are related to the radiotherapy modalities (high total dose, high dose per fraction, large irradiated volume) and to the treatment combinations (e.g., endocrine therapy, chemotherapy, history of surgery) (reviewed in [6]).

Ionizing radiation blocks cells in the G0/G1 and G2/M phases of the cell cycle, and this effect is inhibited by estradiol [7, 8]. Incubation of breast cancer cells with TAM or AIs, such as letrozole, promotes their accumulation in the G0/G1 phase [9, 10]. Moreover, TAM and radiotherapy increase the secretion of pro-fibrotic cytokines and transforming growth factor-beta (TGF-β), a key mediator of fibrogenesis [11, 12]. On the basis of these preclinical observations, a clinical study assessed TAM pro-fibrotic properties in patients with breast cancer and showed that TAM increases the risk of lung fibrosis when given concomitantly with radiotherapy [13]. Similarly, an increased risk of lung fibrosis was also observed in animal models treated concomitantly with TAM and ionizing radiation [14]. However, other preclinical results suggest that TAM could have anti-fibrotic effects by inhibiting TGF-β1-mediated activation of fibroblasts through modulation of SMAD [15] and non-SMAD signaling pathways [16].

Patients- and treatments-related risk factors give a general trend of the probability to develop late radio-induced toxicities. A rapid and non-invasive predictive assay to identify hyper-reactive patients (i.e., patients at high risk of developing late radio-induced toxicity) is now available. This test is based on the evaluation by flow cytometry of radiation-induced CD8+ T-lymphocyte apoptosis (RILA). We previously demonstrated that patients with low RILA scores have a higher risk of severe breast fibrosis [17]. Moreover, in a longitudinal data repository study, we showed that RILA and treatment with TAM are two independent predictive factors of subcutaneous fibrosis in patients with breast cancer [18]. Specifically, in patients with low RILA scores or treated with TAM, the complication (grade 2 or more fibrosis)-free survival rate at 2 years was lower than in patients who did not receive TAM or with high RILA scores.

Therefore, here, we used data we collected during a prospective, multicenter French trial [17] to assess the risk of severe breast fibrosis occurrence in patients with breast cancer who underwent breast-conserving surgery, in function of the adjuvant hormonotherapy (TAM or AI) and individual radiosensitivity, monitored by RILA.

## RESULTS

### Patients’ characteristics

As reported in 2015 [17], 456 patients with breast cancer received radiotherapy and had an evaluable blood sample. All patients underwent breast-conserving surgery followed by whole-breast radiotherapy (*n* = 456), tumor bed boost irradiation (*n* = 449), and lymph node radiotherapy (*n* = 108). Moreover, 143 patients (31.4%) received adjuvant chemotherapy, and 349 (76.5%) were treated with HT (TAM: *n* = 135; AI: *n* = 214) (Table 1).

**Table 1:** Characteristics and treatments of patients divided according to the use or not of hormonotherapy (tamoxifen, aromatase inhibitors, or none)

		No hormonotherapy	Tamoxifen	Aromatase inhibitors	
		*N* = 107 (%)	*N* = 135 (%)	*N* = 214 (%)	*P*-value^*^
Median age (years, range)		56 (29–77)	49 (32–77)	62 (42–88)	<0.001
Fibrosis					
	No	101 (94.4)	114 (84.4)	180 (84.1)	0.02
	Yes	6 (5.6)	21 (15.6)	34 (15.9)	
RILA					
	<12%	47 (43.9)	49 (36.3)	73 (34.1)	0.13
	12–20%	35 (32.7)	44 (32.6)	61 (28.5)	
	≥20%	25 (23.4)	42 (31.1)	80 (37.4)	
Tobacco smoking					
	Non smoker	76 (71.0)	77 (57.0)	136 (63.5)	0.15
	Active/former smoker	29 (27.1)	52 (38.5)	66 (30.8)	
	NA	2 (1.9)	6 (4.4)	12 (5.6)	
Menopausal status					
	Premenopausal	33 (30.8)	92 (68.1)	14 (6.5)	<0.001
	Postmenopausal	71 (66.4)	41 (30.4)	200 (93.5)	
	NA	3 (2.8)	2 (1.5)	0	
Breast volume				
	Small	40 (37.4)	45 (33.3)	50 (23.4)	0.06
	Large	53 (49.5)	68 (50.4)	120 (56.1)	
	NA	14 (13.1)	22 (16.3)	44 (20.5)	
T stage					
	0	0	1 (0.7)	4 (1.9)	0.09
	1	96 (89.7)	112 (83.0)	182 (85.1)	
	2	9 (8.4)	22 (16.3)	28 (13.1)	
	NA	2 (1.9)	0	0	
N stage					
	0	100 (93.5)	109 (80.7)	180 (84.1)	0.01
	1	6 (5.6)	25 (18.5)	28 (13.1)	
	2	0	1 (0.7)	5 (2.3)	
	3	0	0	1 (0.5)	
	NA	1 (0.9)	0	0	
Type of initial surgery				
	Tumorectomy	93 (86.9)	108 (80.0)	180 (84.1)	0.35
	Quadrantectomy	14 (13.1)	27 (20.0)	34 (15.9)	
Margins				
	Negative	105 (98.1)	129 (95.6)	207 (96.7)	0.37
	Positive	1 (0.9)	6 (4.4)	6 (2.8)	
	NA	1 (0.9)	0	1 (0.5)	
Surgical area					
	<50 cm^3^	57 (53.3)	72 (53.3)	104 (48.6)	0.68
	≥50 cm^3^	49 (45.8)	60 (44.4)	108 (50.5)	
	NA	1 (0.9)	3 (2.2)	2 (0.9)	
Adjuvant chemotherapy				
	No	71 (66.4)	86 (63.7)	156 (72.9)	0.16
	Yes	36 (33.6)	49 (36.3)	58 (27.1)	
Adjuvant trastuzumab				
	No	100 (93.5)	130 (96.3)	209 (97.7)	0.17
	Yes	7 (6.5)	5 (3.7)	5 (2.3)	
Node irradiation					
	Mammary gland only	90 (84.1)	94 (69.6)	164 (76.6)	0.03
	Supraclavicular ± internal mammary chain	17 (15.9)	41 (30.4)	50 (23.4)	
Boost					
	No	3 (2.8)	2 (1.5)	2 (0.9)	0.43
	Yes	104 (97.2)	133 (98.5)	212 (99.1)	
Boost technique					
	Photon	78 (72.9)	90 (66.7)	172 (80.4)	0.03
	Electron	13 (12.2)	25 (18.5)	17 (7.9)	
	Brachytherapy	0	1 (0.7)	0	
	Photon + electron	13 (12.1)	14 (10.4)	23 (10.7)	
	NA	3 (2.8)	5 (3.7)	2 (0.9)	

^*^Kruskal–Wallis and Fisher’s Exact tests, NA: non-available.

The incidence of grade ≥2 fibrosis was significantly higher in HT^+^ than in HT^–^patients (15.6% and 15.9% for TAM and AI, respectively, versus 5.6% for HT^–^ patients; *p* = 0.018) (Table 1). RILA score, tobacco smoking and breast volume were not significantly different in the HT subgroups (HT^–^, TAM or AI). No difference was also observed concerning the surgery type/margins and adjuvant systemic therapies (chemotherapy ± trastuzumab). As nodal involvement was significantly higher in the HT^+^ than in HT^–^ group (*p* = 0.01), more patients in the TAM and AI subgroups underwent lymph node irradiation than in the HT^–^ group (*p* = 0.03). The tumor bed boost irradiation techniques were significantly different in the HT^+^ and HT^–^ subgroups: electron beams were more frequently used in the TAM than in the AI group (*p* = 0.03) (Table 1).

### Risk of breast fibrosis according to RILA and HT

This prospective and multicenter French trial reported a 3-year BFFS rate of 87.8% [95% CI 84.4–90.5] [17]. Adjuvant HT and RILA were the two independent factors for breast fibrosis relapse-free survival when adjusted for tobacco smoking (HR = 3.17 [95% CI 1.36–7.39], *p* = 0.008 for HT; and HR = 0.45 [95% CI 0.27–0.74] (*p* = 0.002) for RILA).

**Figure 1:**
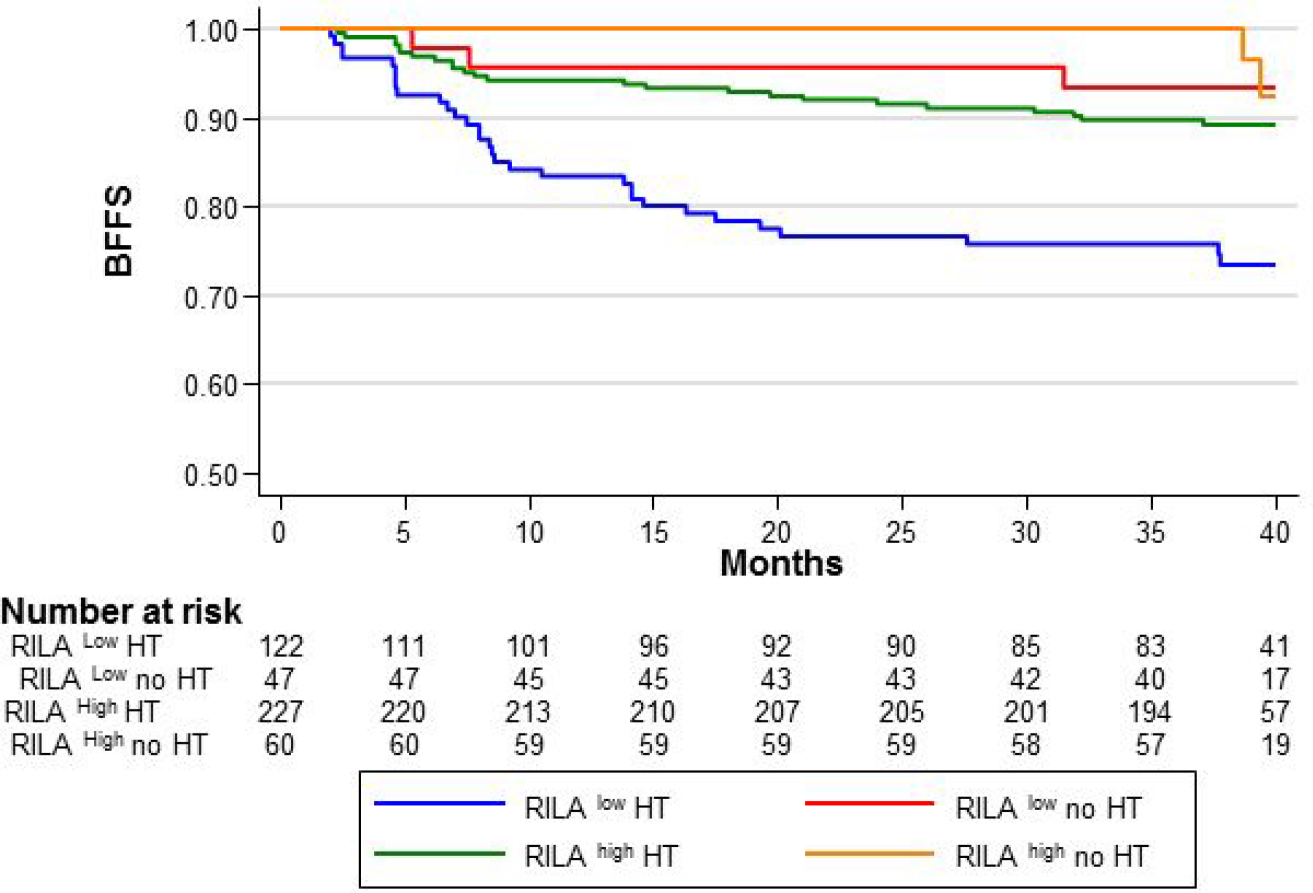
Breast fibrosis-free survival (BFFS) according to the RILA score (<12%, RILA^LOW^ and ≥12%, RILA^HIGH^) and hormonotherapy (with HT, HT; or without, no HT)

Here, we further studied the relationship between RILA and adjuvant HT in grade ≥2 breast fibrosis occurrence (Figure 1). Compared with the reference category (RILA^high^/HT^–^: BFFS=100%), the 36-month BFFS rate was lower in patients with RILA^low^/HT^+^ (75.8%, HR = 5.85 [95% CI 1.79–19.13], *p* = 0.04), with RILA^low^/HT^–^ (93.5%, HR = 1.31 [95% CI 0.26–6.49], *P* = NS) and with RILA^high^/HT^+^ (89.8%, HR = 2.23 [95% CI 0.67–7.40], *p* = NS).

### Risk of grade ≥ 2 breast fibrosis in the TAM and AI groups

**Figure 2:**
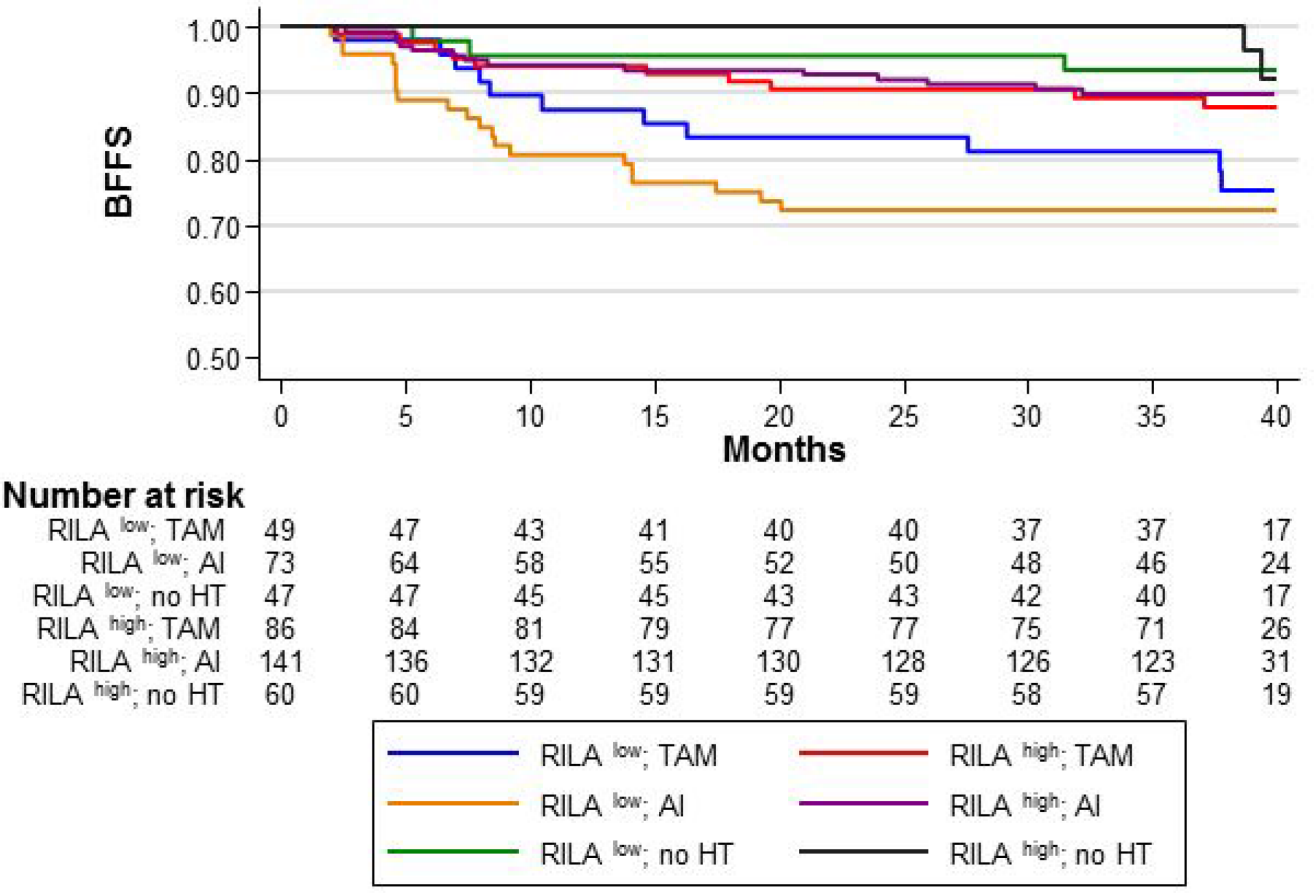
BFFS according to the RILA score (RILA^LOW^ or RILA^HIGH^) and the HT sub-categories: “TAM/no TAM”, “AI/no AI”, and “no HT”

Compared with the reference category (RILA^high^/HT^–^), in RILA^low^ patients, adjuvant TAM or AI significantly increased the risk of severe breast fibrosis (HR = 3.81 [95% CI 1.06–13.66], *p* = 0.04; and HR = 5.02 [95% CI 1.49–16.92], *p* = 0.009, respectively), without significant difference between TAM and AI (*p* = 0.46) (Figure 2). The 36-month BFFS rates were 81.2% and 72.2% in the TAM and AI groups, respectively.

In RILA^high^ patients, adjuvant HT slightly increased the risk of severe breast fibrosis (HR = 2.43 for TAM [95% CI 0.67–8.88, *p* = 0.177] and HR = 2.12 for AI [95% CI 0.61–7.40, *p* = 0.236], without significant differences (Figure 2). The 36-month BFFS was 89.5% (TAM) and 90% (AI) for HT^+^ patients compared with the reference category (RILA^high^/HT^–^).

### Risk of breast fibrosis according to HT timing (co-HT or sq-HT)

**Figure 3:**
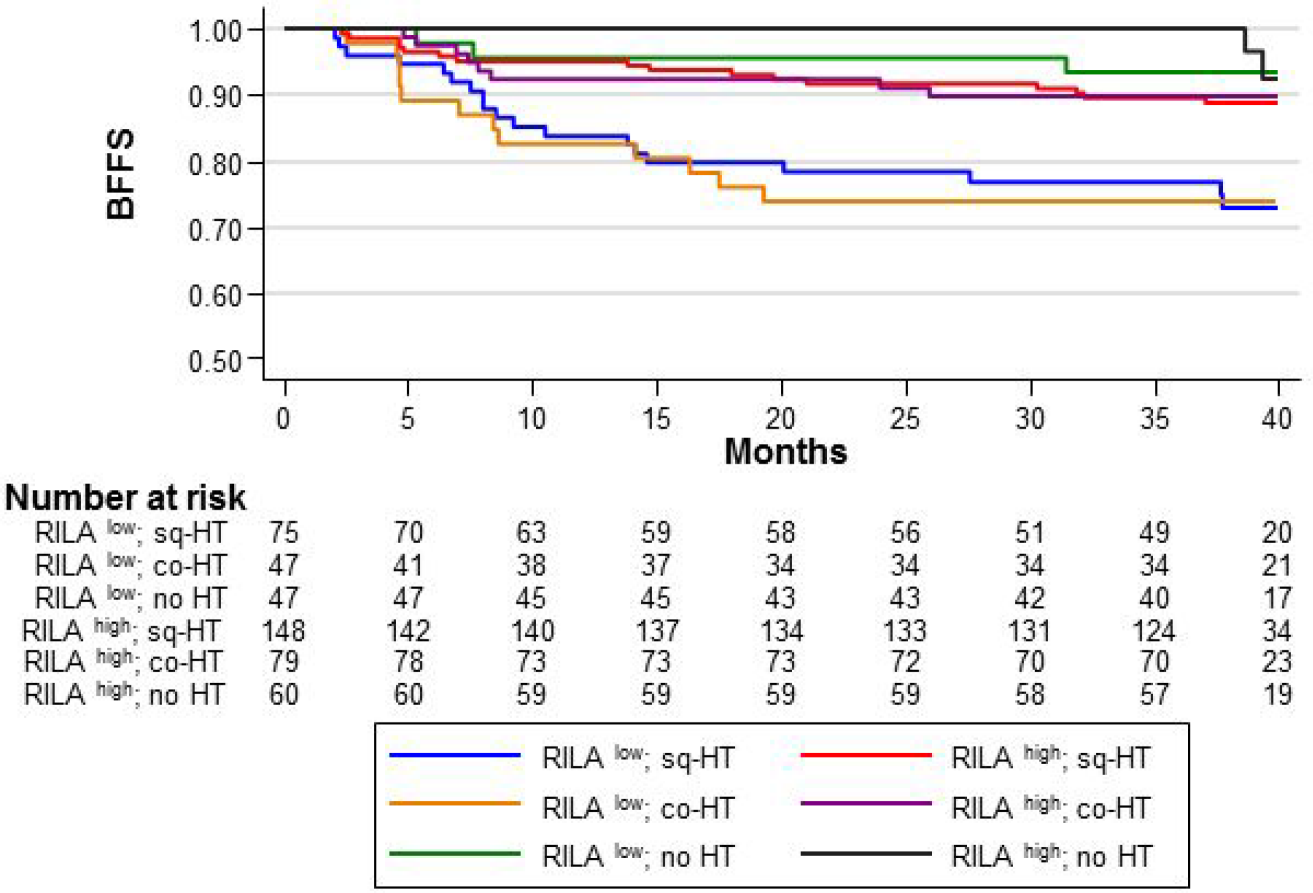
BFFS according to the RILA score (RILA^LOW^ or RILA^HIGH^) and HT timing relative to radiotherapy: concomitant “co-HT”, sequential “sq-HT”, or none “no HT”

Compared with the reference category (RILA^high^/HT^–^), in RILA^low^ patients, the 36-month BFFS rate was 73.9% in the co-HT and 76.9% in the sq-HT group without significant differences between groups (Figure 3). Both co-HT and sq-HT increased the risk of severe fibrosis (HR = 4.47 [95% CI 1.32–15.12], *p* = 0.016 and HR = 4.58 [95% CI 1.29–16.25], *p* = 0.018, for the co-HT and sq-HT group, respectively).

Similar results were obtained for RILA^high^/HT^+^ patients (Figure 3). The 36-month BFFS rates were 89.9% and 89.7% in the co-HT and sq-HT group, respectively. However, HT timing did not increase the risk of severe fibrosis (HR = 2.05 [95% CI 0.54–7.76], *p* = 0.288 and HR = 2.35 [95% CI 0.69–8.07], *p* = 0.174, for co-HT and sq-HT, respectively).

## DISCUSSION

In the present study, data on late toxicities from a prospective and multicenter French study on patients with breast cancer were used to assess the interactions between HT and radiotherapy in breast fibrosis occurrence. Our results showed that patients with low RILA score and treated with HT had the highest risk of severe late side effects.

A large and exhaustive literature review was recently published and reported no difference in esthetic outcome and toxicities in patients with breast cancer who underwent sequential or concomitant endocrine treatments [19]. TAM use and timing, relative to radiotherapy, were studied in three retrospective studies with a very long follow-up (>8 years) [20–22]. Pierce and colleagues reported a subgroup analysis of the Intergroup 0102 phase III randomized trial. Only grade 3 or more late toxicities were reviewed and no difference of lung toxicity was observed in patients who received concurrent and sequential TAM use [22]. The monocentric and retrospective study by Harris and colleagues also did not observe any difference in terms of late side effects (breast edema, arm edema, rib fractures, pneumonitis, 3- and 5-year cosmesis) between concurrent and sequential TAM use [21]. Similar findings were reported for concurrent and sequential AI use in two retrospective cohorts [23, 24] and in a randomized phase II clinical trial (Concomitant HOrmono-RadioTherapy, CO-HO-RT, NCT00208273) that investigated the timing of endocrine therapy and radiotherapy in patients with breast cancer [25]. The long-term follow-up of the CO-HO-RT trial did not report any statistical difference regarding HT timing, but its translational sub-studies showed that low RILA values (used as a stratification factor) were associated with a higher risk of breast fibrosis [26]. Our present results confirm that HT timing does not affect the risk of breast fibrosis. In line with the CO-HO-RT results, we also observed that low RILA values were associated with higher risk of breast fibrosis, regardless of HT timing. More precisely, the association of low RILA score and HT significantly increased the risk of breast fibrosis by 5- to 6-fold. These results confirm our previous finding that TAM enhances breast fibrosis risk only in hypersensitive patients (i.e., patients with low RILA scores) [18].

While no data has been published on the molecular mechanisms involved in AI-related fibrosis, conflicting results have been reported about TAM role in fibrosis occurrence. Recent *in vitro* results showed that TAM prevents fibroblast activation by TGF-β as effectively as a TGF-β receptor kinase inhibitor (GW6604) [16]. Similarly, in another *in vivo* model (renal tubulointerstitial fibrosis), TAM could reduce renal fibrosis by decreasing the expression of extracellular matrix proteins and tissue TGF-β [15]. On the other hand, Bese and Yavas [14, 27] observed that the concomitant use of TAM, but not AI with radiotherapy increased radiation-induced pulmonary toxicity. However, they did not study the underlying mechanism of action. Other authors reported that the blood plasma level of TGF-β-1 was comparable between patients with breast cancer who developed or not lung fibrosis [28]. Moreover, they did not find any difference in lung fibrosis occurrence in patients who received TAM before or concomitantly with radiotherapy. Finally, Ryu and colleagues [29] showed that in a liver fibrosis rat model, TAM decreases both plasma and tissue TGF β-1 expression.

In conclusion, we showed that patients with low RILA scores are at higher risk of developing radiation-induced fibrosis [17]. Moreover, in these patients, HT further increased this risk, although preclinical data reported that TAM has an anti-fibrotic effect. Therefore, it is now important to study the molecular mechanisms to understand why HT significantly increases the risk of fibrosis only in patients identified as hypersensitive to radiotherapy.

On the basis of these results, a patent was filed for determining the risk of developing fibrosis in the clinical practice (patent No. PCT/EP2017/071887).

## MATERIALS AND METHODS

### Study design, patients’ selection and treatment

This French prospective multicenter study (NCT00893035) enrolled 502 patients to assess RILA as a predictive tool of breast fibrosis after adjuvant breast radiotherapy [17]. Two treatment groups were characterized: patients who received adjuvant hormonotherapy (HT^+^) and patients who did not (HT^–^). HT^+^ patients were then divided in two subcategories (treated with TAM or AI) to study the effect of each pharmacological class on the risk of grade ≥2 fibrosis. Moreover, hormonotherapy (HT) was defined as concomitant (co-HT) with radiotherapy when started before or the same day as radiotherapy, and sequential (sq-HT) when started after the last day of radiotherapy.

### Toxicity assessment

Toxicities were prospectively evaluated at baseline, every week during radiotherapy, one, three and six months after radiotherapy completion and then every six months up to month 36. Cutaneous and subcutaneous toxicities were assessed using all the possible definitions described in the CTCAE v3.0 grading scale for the Dermatology/Skin category. Grade ≥2 breast fibrosis (primary endpoint) was blindly scored by at least two physicians.

### Radiation-induced lymphocyte apoptosis (RILA) [30]

Briefly, 200 µl of heparinized whole blood was cultured for 24 hours and then irradiated with 8 Gy or not (control samples). After 24 hours, CD8+ lymphocytes were separated from the other blood cells and their apoptosis percentage was assessed by flow cytometry. RILA was defined as the population of CD8+ lymphocytes with reduced DNA fluorescence and calculated as the percentage of total T-lymphocyte death induced by irradiation (8 Gy) minus the spontaneous cell death (0 Gy) [17].

### Statistical analyses

Categorical variables were described as frequencies and percentages and continuous variables as medians and ranges. Comparisons between groups were performed with the Fisher’s Exact and Kruskal–Wallis tests, respectively. Absolute changes in RILA scores before and after irradiation were evaluated as categorical variables. Three categories were constructed around the 33% quantile (<12, 12–20, and ≥20) and then merged in two categories, leading to two main subcategories: RILA low (RILA^low^) and RILA high (RILA^high^) for values lower and higher than 12%, respectively. Breast Fibrosis-Free Survival (BFFS) was defined as the interval between the radiotherapy start and the occurrence of a grade ≥2 breast fibrosis. Patients alive who never experienced a grade ≥2 breast fibrosis at the last follow-up were censored. BFFS rates were estimated using the Kaplan–Meier method. Ninety-five percent confidence intervals (95%CI) were also determined.

The effect size was estimated by univariate analysis using the Cox proportional hazard regression model. Overall comparisons were performed using the log-rank test, and subgroup effect size comparisons with the Wald test. The median follow-up was estimated with the inverse Kaplan–Meier method. A *P*-value < 0.05 was considered as significant. All statistical tests were two-sided. Analyses were carried out with the Stata software, v13.

## Abbreviations

TAM: tamoxifen
AI: aromatase inhibitors
RILA: radiation-induced CD8+ T-lymphocyte apoptosis assay
HT^+^: patients group who was treated by adjuvant hormonotherapy
HT^–^: patients group who was not treated by adjuvant hormonotherapy
co-HT: concomitant hormonotherapy
sq-HT: sequential hormonotherapy
RILA^low^: RILA value lower than 12%
RILA^high^: RILA value higher than 12%
BFFS: Breast Fibrosis-Free Survival
95% CI: ninety-five percent confidence intervals
